# Analysis of Climate and Income-Related Factors for High Regional Child Drowning Mortality in China

**DOI:** 10.3389/ijph.2022.1604882

**Published:** 2022-06-02

**Authors:** Yi Huang, Hujing Shi, Xiaoxiao Liu, Xianjing Lu, Jin Zhang

**Affiliations:** ^1^ School of Geographic Sciences, Nantong University, Nantong, China; ^2^ State Key Laboratory of Hydrology Water Resources and Hydraulic Engineering, Yangtze Institute for Conservation and Development, Hohai University, Nanjing, China; ^3^ Xinjiang Institute of Ecology and Geography, Chinese Academy of Sciences, Urumqi, China

**Keywords:** temperature, precipitation, southern China, northern China, climate, child drowning

## Abstract

**Objectives:** To assess the relationship between regional climatic factors and child drowning in China.

**Methods:** Provincial age-specific drowning rate, climatic and income data were collected. We conducted a geographically weighted regression to evaluate the association between drowning and climatic factors. A generalized additive model was used to comprise a bivariate term with which to investigate the interaction of environmental risk factors and whether such interactions influence drowning mortality.

**Results:** In southern China, an abundance of water systems and increased precipitation, as well as hotter and longer summers, lead to significantly higher drowning compared with that in northern China. Long summers and low economic performance in parts of Xinjiang were key factors for its high drowning mortality rate. Linear and nonlinear joint effects were observed between the risk factors of drowning.

**Conclusion:** Different regions should use adaptive measures to reduce drowning risks, for example, communication campaigns during the summer period or when the weather changes.

## Introduction

The geographical distribution of climatic factors, e.g., temperature, air pressure, precipitation and flooding, air quality and contamination, and humidity, can strongly influence the health, mortality, and longevity of humans. To date, studies have mainly focused on the direct influence of climate on disease and mortality, especially in terms of temperature-related diseases (e.g., cardiovascular and respiratory diseases) that mostly occur in the elderly. However, climate can have an indirect impact on mortality, i.e., that which is not caused by disease. Drowning is a leading cause of death in young people, especially in low- and middle-income countries [1]. Current studies on drowning have concentrated on epidemiological characteristics (e.g., age, sex, and position) [2–4], clinical treatment [5], and social risk factors (e.g., poverty, parental education, full-time or part-time care status, safety education, etc.) [6, 7]. Aside from social factors related to drowning that would change with economic performance, natural risk factors could prove to be decisive in relation to drowning rates among regions with similar development stages.

The main objectives of the present study were as follows: 1) to determine regional differences of child drowning between southern China and northern China; 2) to assess the relationship between regional climatic factors and child drowning in China and provide corresponding suggestions based on the findings; and 3) calculate the life expectancy lost to drowning in China.

## Methods

### Data Source

Mainland China has 31 provinces, with the north–south boundary of China being the Qingling Mountain and Huai River; southern and northern China each contain 15 provinces. Tibet is not included in either northern or southern China because the average elevation in Tibet is > 4,500 m and its temperature is lower than that of most of the northern provinces of China. The provinces of northern and southern China are illustrated in Supplementary File S1.

#### Age-Specific Drowning Mortality Rate

About 63,724 persons died from unintentional drowning in China in 2016, accounting for 21% of global drowning deaths [8]. We collected age-specific mortality rates associated with drowning in China in 1990 and 2013 from the national disease monitoring system of China, national maternal and child health monitoring network, China CDC (Centers for Disease Control and Prevention) death cause registration and reporting information system, and a related study [9].

#### Monthly Mortality Data for Young People in China

As the first cause of death for 1–14-year-olds, drowning contributed half of all-cause mortality in this age group in 1990. Drowning is closely linked to season; thus, we also collected monthly mortality data for young people in China from the fourth China population census data collected in 1990.

#### Data on Precipitation, Temperature, Per Capita Income, Population Density, and Water Systems

To explore why the drowning mortality rate is much higher in southern China than that in northern China, and to determine why Xinjiang in northern China has the highest drowning mortality rate, we collected data on relevant factors that might influence drowning. Most drowning incidents occur in natural waters and man-made water bodies in natural settings (e.g., ponds, canals, streams, rivers, reservoirs, lakes, etc.) or agricultural/irrigation water sources [10, 11]. In addition, living close to water is an important risk factor for drowning (especially in rural areas), with 71.6% of those that died from drowning having lived within 100 m of a body of water; moreover, fatal drowning mainly occurs in summer, which may be because temperature is higher and children are more likely to swim [12]. A previous study found that high temperature and abundant rainfall both increase the probability of drowning, and that children who regularly swim in or play near water have a higher risk of fatal drowning [13]. In summary, precipitation, proximity to a water system, per capita income, temperature in summer, and the duration of summer were selected as variables to further investigate the regional differences in drowning in China.

For the above mentioned reasons, six types of data were collected. From the China Meteorological Data Network (http://data.cma.cn), we collected spatial data of precipitation, temperature in July, and the number of days for which the mean temperature was >25°C in 1990 and 2013. Daily temperature data in 1990 and 2013 were collected from 839 temperature monitoring stations across the country. Per capita income data (US dollar) were obtained from the China and Provincial Statistical Yearbook (http://www.stats.gov.cn/tjsj/ndsj/). In addition, a 1 × 1 km population density grid map and a map of river and lake systems were collected from the Resource and Environment Science and Data Center (http://www.resdc.cn) owned by the Chinese Academy of Sciences; data on 110,596 rivers and 134,650 lakes/reservoirs were collected.

### Analysis Methods

#### Normality Tests and T-Tests

The normal distribution and t-test were performed to identify statistical differences in drowning and climatic factors between northern and southern China.

#### Calculation of Proximity to a Water System

The proximity to water system was determined using three parameters: water system density (including rivers, lakes, reservoirs, etc.), population density, and the distance between a residential area and water system. Kernel density was used to calculate the density of lakes and reservoirs, whereas line density was used to calculate the density of rivers. Overall, the proximity to a water system was calculated according to Eq. 1.

$$PW_{i}=P_{i}\times K_{i}\times L_{i}$$ (1)

where *PW*_*i*_ is the proximity to a water system in grid *i* (1 × 1 km), *P*_*i*_ is the population density in grid *i*, *K*_*i*_ is the kernel density of lakes and reservoirs in grid *i*, and *L*_*i*_ is the line density of rivers in grid *i*.

#### Geographically Weighted Regression

Geographically weighted regression (GWR) was used to analyze the geographical regression between drowning mortality and climatic factors from a spatial perspective. The estimate value (EV) and *p*-value were calculated to evaluate the effect of climatic factors on drowning mortality. Akaike information criterion (AIC), AIC corrected, Schwarz’s Bayesian information criterion (BIC), and adjusted R^2^ values were calculated to evaluate the effectiveness of GWR.

#### Generalized Additive Model

Generalized additive models (GAMs) are flexible models used to explore the complex nonlinear associations among independent and dependent variables. In this study, a GAM was used to comprise a bivariate term with which to investigate whether climatic and nonclimatic factors interact and whether such interactions influence drowning mortality rate.

A nonparametric binary response model was constructed using a thin-plate spline function for qualitative analysis, and the interaction between each climatic and nonclimatic factor was constructed. The predicted results of interactions related to drowning mortality were displayed in a three-dimensional surface graph:

$$\text{Log}\left[\text{E}\left(Y\right)\right]=\text{α}+TS\left(x_{i},\;x_{j}\right)+S\left(x_{i}\right)+S\left(x_{j}\right)$$ (2)

where Y is the drowning morality rate, *α* is the intercept*,* TS is the thin-plate spline, and *S* is the smoothing function.

#### Calculation of Life Expectancy Reduction Caused by Drowning: Comparing Northern and Southern China

Life expectancy refers to the amount of life remaining at birth, which is an estimate of the average expected lifespan under certain conditions according to current mortality rates; life expectancy is calculated using age-specific mortality rate. The life expectancy lost due to drowning was calculated and compared between northern and southern China.

## Results

### Spatial Distribution of Drowning and Climatic Factors

**FIGURE 1:**
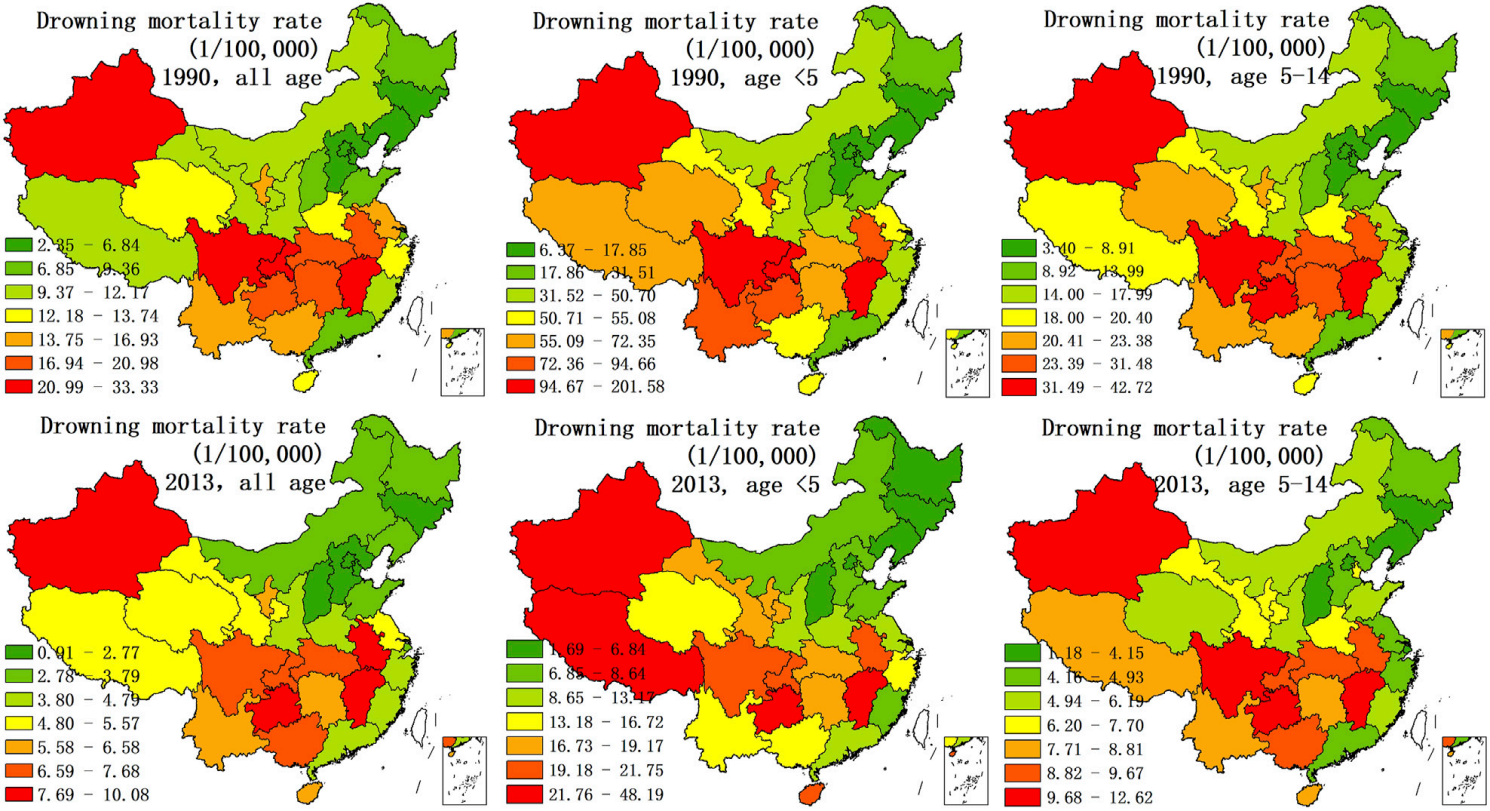
Distribution of standardized and age-specific mortality rates of drowning (1/100,000) (China, 1990 and 2013).

Figure 1 shows the spatial characteristics of drowning in China. The mortality rate for drowning was higher in southern China than that in northern China, with increased precipitation and a higher density of river and lake systems, especially in inland less-developed regions, suggesting that precipitation, water systems, and economic performance are crucial factors in drowning rate. Indeed, the second to tenth rankings for drowning mortality among 31 provinces were located in the south. In addition, the drowning mortality rate in Xinjiang Uygur Autonomous Region (located in northwestern Chia) was ranked first in China, despite the rarity of precipitation in Xinjiang.

Supplementary File S2 shows the monthly proportion of death in China according to age. Young people in China were more likely to die in summer, especially in July (the month with the highest temperature) followed by June and August. As the first cause of mortality of young Chinese people, drowning was clearly associated with temperature.

Supplementary File S3 shows the mean half-month temperature from May to October in different zones. According to this data, summer in the marginal tropics and south subtropical zone is longer than 6 months every year; thus, the temperature is suitable for swimming and water-related play from May to October. Summer in south Xinjiang and the mid-north subtropical zone (Yangzi river basin) can last for four to 5 months; although the summer period is shorter, the temperature in July and August is usually higher compared with that in the south subtropical zone.

**FIGURE 2:**
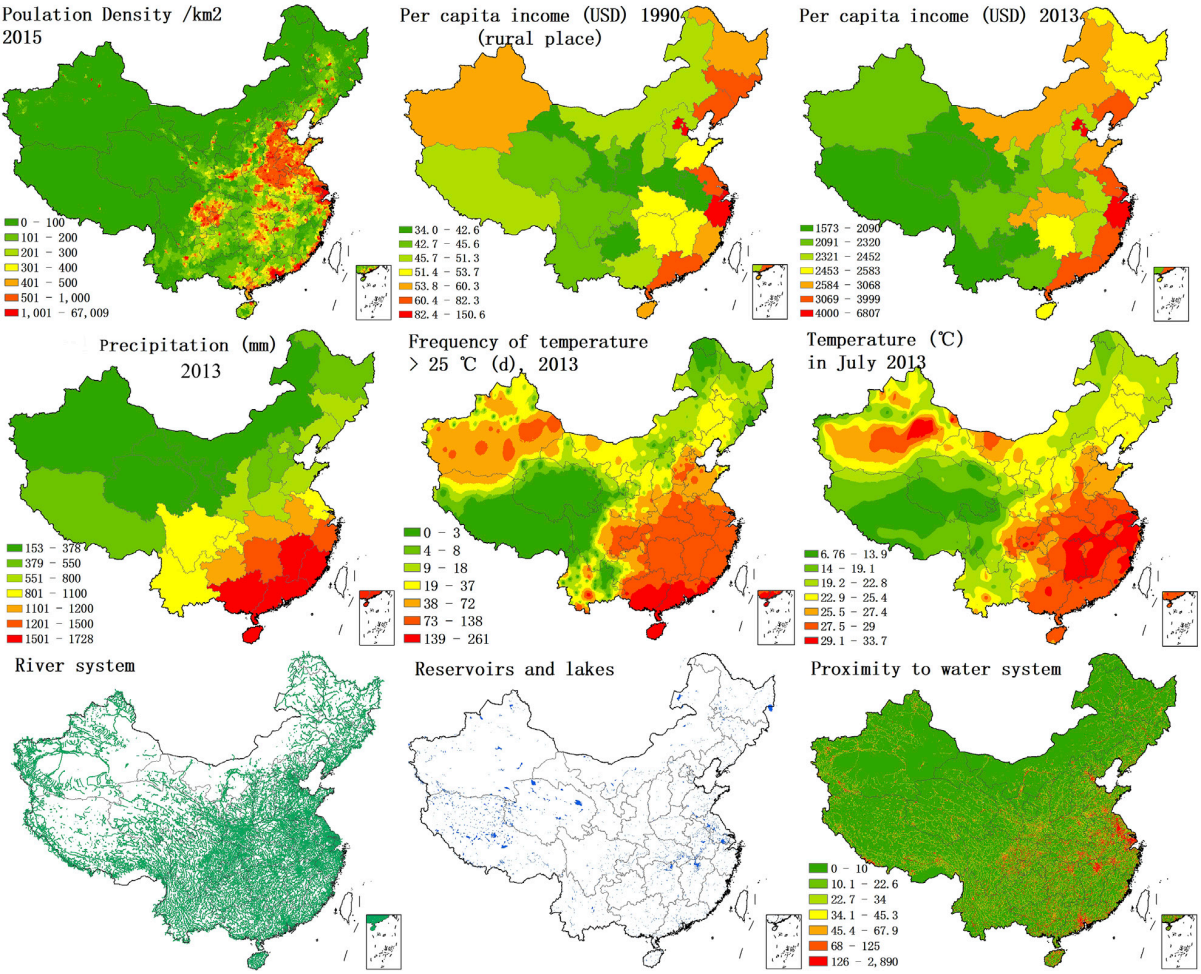
Distribution of provincial precipitation, temperature in July, duration of summer, population density, water systems, and per capita income (China, 2013 and 2015).

Figure 2 shows the distribution of provincial precipitation, temperature in July, number of days for mean temperature was >25°C, population density, river systems, lake and reservoir systems, and per capita income in China. Precipitation levels decreased from southeastern China to northwestern China, whereas the spatial characteristics of temperature in July were not entirely consistent with precipitation. The highest temperature in July was found not in southern China but rather in south-central China (Hunan province, Jiangxi province, Fujian province, and Zhejiang province), which is covered by the subtropical high and has several days in July (almost every year) for which the mean temperature is > 35°C. Another area in which summer is hot in the southern Xinjiang Uygur Autonomous Region located in northwestern inland China; this is due to its special terrain and underlying surface. The density of water systems was found to be higher in southern China than that in northern China. Precipitation is the main water source for rivers and lakes, and plentiful precipitation shapes the development of water systems in southern China, especially those in the Yangzi river basin. However, no significant north–south difference existed for per capita income in China, although the coastal regions, both in the north and south, had higher per capita income than that in the central and western regions.

### Statistical Differences in Drowning and Climatic Factors Between Northern and Southern China

Normality tests and t-tests were performed to compare drowning mortality rates, precipitation, temperature, and income between northern and southern China (Table 1). Per capita income was balanced between northern and southern China; however, drowning mortality rate, precipitation levels, temperature, and proximity to water were statistically lower in northern China relative to those in southern China.

**TABLE 1:** Drowning, precipitation, temperature, and income compared between northern and southern China (China, 1990 and 2013).

	Drowning 1990	Drowning 1990, 1–4	Drowning 1990, 5–14	Drowning 2013	Drowning 2013, 1–4	Drowning 2013, 5–14
T-value	−3.453	−2.825	−2.359	−3.552	−1.456	−2.352
*p*-value	0.002**	0.009**	0.026*	0.001***	0.157	0.026*
	**Precipitation 2013**	**Temperature In July 2013**	**Income 1990**	**Income 2013**	**Temperature >25°C 2013**	**proximity to water 2015**
T-value	−10.317	−4.091	−0.465	-0.722	−4.845	−2.396
*p*-value	0.000***	0.000***	0.537	0.283	0.000***	0.024*

*Significant at *p* < 0.05 level, ** significant at *p* < 0.01 level, *** significant at *p* < 0.001 level.

### Relationships Between Multiple Factors and Drowning

**FIGURE 3:**
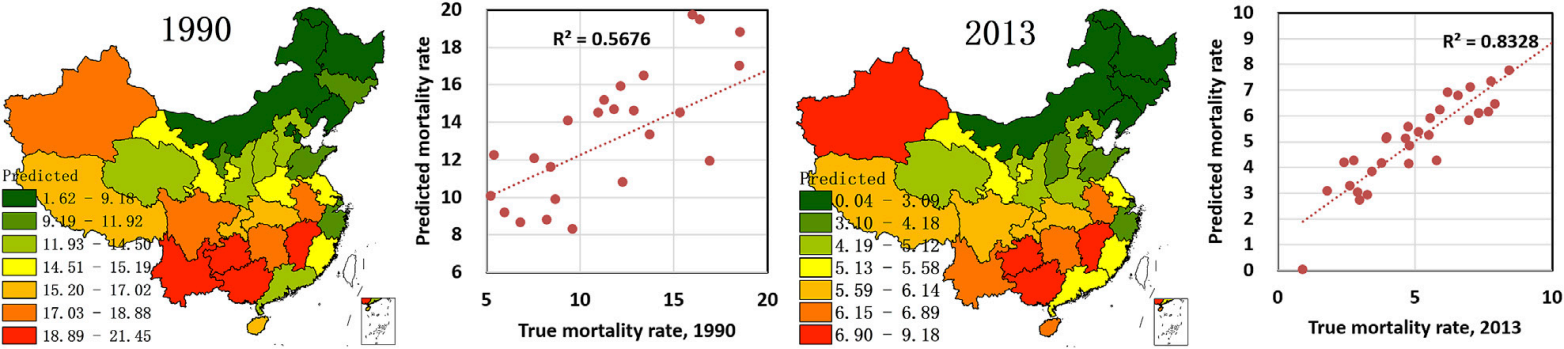
Simulated drowning mortality rate in China with climatic factors (precipitation, proximity to water, frequency of temperature >25°C, and per capita income) (China, 1990 and 2013).

To quantitatively assess the relationship between climatic/comprehensive factors and drowning, precipitation, proximity to water system, temperature in July, frequency of temperature >25°C, and per capita income were chosen as independent variables, whereas drowning mortality rates in 1990 and 2013 were chosen as dependent variables. A GWR of factors and drowning mortality rate was conducted (Table 2), and the predicted value of the regional drowning mortality rate with precipitation, proximity to water, frequency of temperature >25°C, and per capita income are shown in Figure 3.

**TABLE 2:** Geographically weighted regression of drowning mortality rate and variables of interest (China, 1990 and 2013).

Indicators	Variable	*EV*	t	*p*	AIC	AICc	BIC	R^2^	A-R^2^
Standard drowning mortality rate, 1990	Precipitation	0.291	2.359	0.018*	115.997	123.064	142.775	0.747	0.687
	proximity to water	−0.095	−0.736	0.462					
	temperature >25°C	0.259	2.074	0.038*					
	per capita income	−0.407	−3.448	0.001***					
	temperature in July	0.186	1.465	0.143					
Standard drowning mortality rate, 2013	precipitation	0.315	2.571	0.010**	69.127	77.023	97.292	0.884	0.854
	proximity to water	−0.105	−0.816	0.415					
	temperature >25°C	0.325	2.665	0.008**					
	per capita income	−0.471	−4.134	0.000***					
	temperature in July	0.146	1.145	0.252					

*Significant at *p* < 0.05 level, ** significant at *p* < 0.01 level, *** significant at *p* < 0.001 level.

**FIGURE 4:**
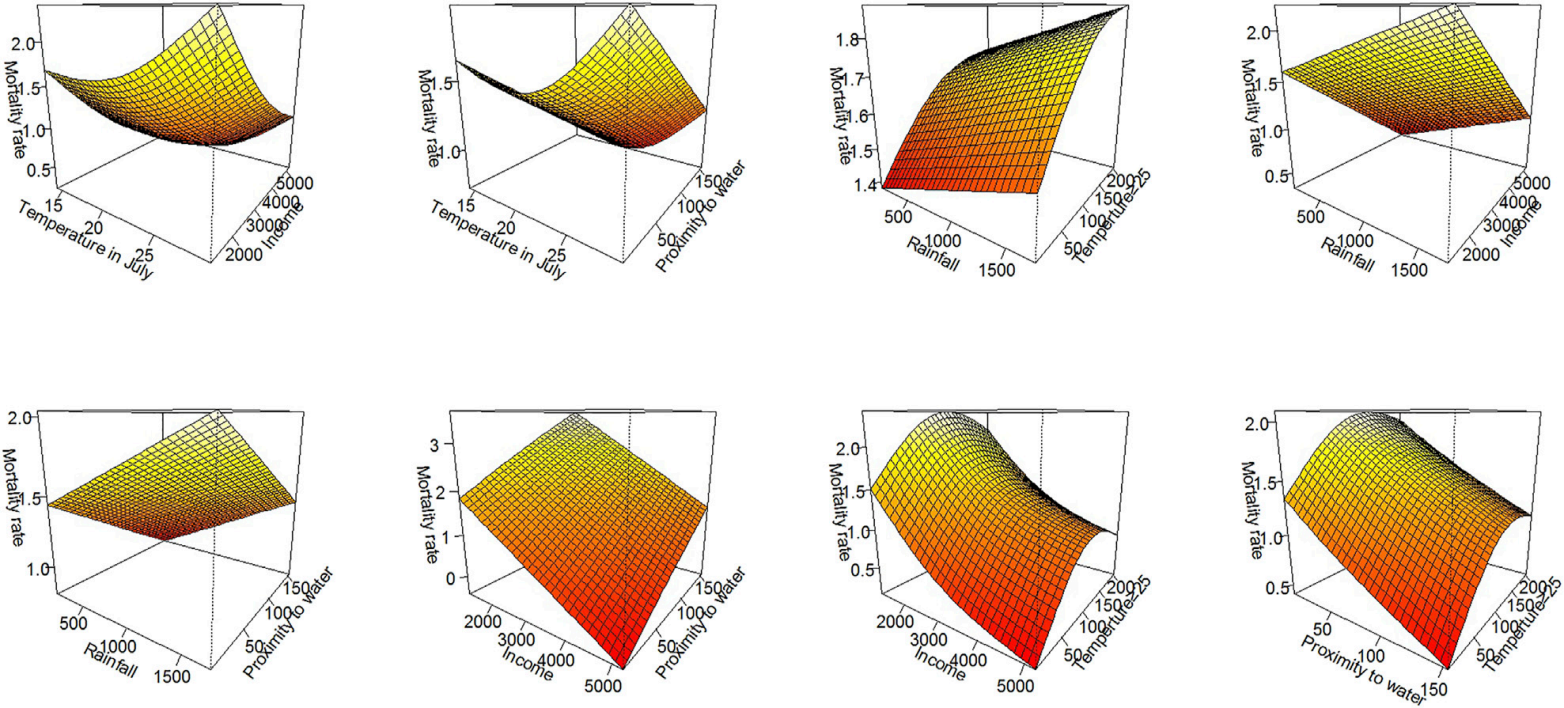
Bivariate response surfaces of each factor and standardized drowning mortality rate (in logarithmic form) (China, 2013).

Figure 4 depicts the interactive effect between each climatic and nonclimatic factor on drowning mortality in 2013 using three-dimensional visualization graphs. Drowning mortality was positively associated with temperature in July, rainfall, and frequency of temperature >25°C, but it was negatively associated with per capita income. Visual inspection revealed that the joint effects between each factor on drowning mortality were complicated. Linear joint effects were observed between rainfall and income, as well as between income and proximity to water. Nonlinear joint effects were observed between other factors.

### Calculation of Life Expectancy Reduction due to Drowning

Life expectancy lost due to drowning-related mortality was 0.245 ± 0.067 years in northern China and 0.492 ± 0.089 years in southern China in 1990; in 2013, the loss of life expectancy fell to 0.101 ± 0.027 years in northern China and 0.162 ± 0.018 years in southern China. A provincial map is provided in Supplementary File S4. An apparent boundary of life expectancy lost due to drowning existed between southern and northern China. The middle stream and upstream of the Yangzi river basin, with well-developed water systems, hot summers, plentiful precipitation, and low income, suffered most from drowning.

## Discussion

To our knowledge, this is the first study in which regional differences in climate were investigated in relation to drowning mortality in China. From 2008 to 2017, drowning was the first major cause of death for young people (≤14 years old) in China according to the China Health Statistics Yearbook; indeed, about one quarter of deaths in 1–14-year-olds were due to drowning.

Precipitation, temperature in summer, duration of summer, river and lake density, and regional development level are all crucial factors related to drowning rate. China has a land range of 4,000 km from the north to the south; thus, its internal climate varies substantially, and its annual precipitation varies from <200 mm in northwestern China to >2,000 mm in southeastern China, with fluctuations of as much as 1,800 mm recorded. Southern China has an abundance of water bodies, whereas northern China has a serious problem with water shortages. In the current study, precipitation was found to be a leading factor in the north–south difference in drowning mortality. Indeed, precipitation and water systems are directly involved in drowning, and precipitation in China is concentrated in summer, especially in the Yangtze River basin in June and July, which is known as the “rainy season.” In addition, southern China usually suffers from typhoons (very violent tropical storms) in summer and autumn that are generated in the tropical Pacific Ocean; the rainy season and typhoons bring short-term heavy rainfall that always lead to floods. Increased water levels and violent water flow in turn increase the proportion of residents that die due to drowning.

Per capita income was also found to be a factor related to drowning, which is in accordance with formal studies on urbanization and drowning: the drowning mortality rate in rural areas and less-developed regions is much higher than that in urban and developed regions. Urbanization could affect the risk of drowning in many ways [14]; for example, it reduces access to natural waters and the need to use them because many swimming pools can be found in cities, and it increases income, which varies inversely with drowning rates [15, 16]. In addition, safety and first aid education in urban areas is superior to that in rural areas, where 83.1% of residents have no knowledge of first aid skills related to drowning [7]. Western China has a lower per capita income and a high drowning mortality, even though some western provinces are not particularly hot and rainy. No statistical differences in per capita income and urbanization rate existed between northern and southern China (Table 1); therefore, they cannot explain the north–south difference in drowning rates.

In China, average temperature in July varies from 19°C in northeastern China to 31°C in the south. Additionally, the duration of summer varies from less than 1 month in northeastern China to 6 months in the south. Swimming is one of the main causes of drowning, and people prefer to swim when temperature is high in summer. In particular, children can often be found playing together in water bodies because the water temperature in southern China is suitable for swimming and play; however, lower atmospheric temperature, which results in lower water temperature, likely reduces the drowning mortality rate. Compared with temperature in July, the duration of summer (here, the period when temperature was >25°C) is more tightly associated with drowning mortality because longer periods in which the temperature is high increase the amount of swimming and water play, which in turn increases drowning risk.

The duration of summer and temperature in July could also explain why the Xinjiang Uygur Autonomous Region is the single Northern Province ranked highly for drowning mortality, unlike the other Northern provinces. The average temperature in July in southeastern Xinjiang can be 33°C because of the unique terrain; thus, the temperature is even higher than that of the hottest province in southern China (Supplementary File S3). In addition, the summer in south Xinjiang is longer than that of half the provinces in southern China, whereas the per capita income is lower than the average in China. In summary, higher temperatures and longer summers increase the probability of swimming/water play, while the poorer economy increases the difficulty in preventing drowning.

The distance between a residence and water system is also an important factor in drowning risk, as young people are more likely to swim if they live near a water system. However, this effect was not statistically significant in the present study. This may be because the resolution of our data is too large to find meaningful results. Drowning incidents usually occur within 100 m of a body of water [7], but the resolution of our data (i.e., the population density) was 1 km; such a distance may be too far for children to find a natural water body.

Children and teenagers are high-risk age groups for drowning because they are curious, lack self-awareness, and are motivated to frequently perform a wide range of activities [14]. The abundance of water resources and higher temperature increases the drowning mortality rate of young people in southern China. As society and the economy develop in China, the mortality rate attributed to drowning will likely decrease in the future; however, drowning mortality will still be higher in southern China than in northern China because of the higher rainfall and temperature in the former.

We can conclude that the spatial heterogeneity of regional drowning mortality can be attributed to comprehensive effects from multiple climate–economy factors. Consequently, it is important for local government to prevent drowning according to the characteristics of the local climate. For example, leaders in southern China (e.g., Guagnxi, Guangdong, and Hainan) should implement defensive measures that last longer because summer lasts as long as 6 months (May–October), whereas those in the Yangzi river basin and south Xinjiang should focus their attention on protection during July and August when the temperature is hottest (usually >30°C). The central government should take more effective measures to mitigate economic inequality in China, which will help ensure that appropriate measures are taken in low-income regions to better respond to drowning risk, especially in western China.

There are some limitations to this study. First, the periods of drowning and risk factors are not entirely consistent. The periods of drowning are also different. For example, age specific drowning rate was in 1990 and 2013, monthly mortality data was collected in 4th China census in 1990, which may have affected the accuracy of the results. Second, ecological bias (fallacy) was inevitable on ecological research methods to determine the relationship between climatic factors and drowning. Third, social factors associated with drowning prevention and control were complex, in addition to income, management of drowning hot spots, child care system and first aid education and other related factors need to be further considered.

### Conclusion

The conclusion drawn from this study are as follows. (1) Drowning mortality rate is statistically higher in southern China than in northern China: drowning resulted in 0.247 and 0.061 years of lost life expectancy in southern China compared with that in northern China in 1990 and 2013, respectively. (2) Precipitation and temperature are key factors in the north–south difference in drowning rate; high temperature in summer, longer summers, and a poorer economy explain the high drowning mortality rate in Xinjiang, despite its location in northwestern China. (3) The duration of summer is more influential on drowning than the temperature in July. (4) Linear and nonlinear joint effects were observed between the risk factors for drowning.

These findings suggest that the government in China should implement positive, efficient, and adaptive steps, especially in conjunction with regional climatic and economic development. Different regions should use adaptive measures to reduce drowning risks, for example, communication campaigns during the summer period or when the weather changes.

## Data Availability

The datasets generated during and/or analyzed during the current study are available at http://data.cma.cn, http://www.stats.gov.cn/tjsj/ndsj/, http://www.resdc.cn, and https://www.cnki.net/.

## Supplementary Material

Supplementary File S1 Map of northern China and southern China.

Supplementary File S2 Age-specific monthly proportion of death (China, 1990) (668,000 mortality).

Supplementary File S3 Mean temperature of different zones every half month (China, 2013). F: first half; S: second half.

Supplementary File S4 Life expectancy lost caused by drowning (year) (China, 1990 and 2013).
