# Supplementary figures and images for: Analysis of Climate and Income-Related Factors for High Regional Child Drowning Mortality in China
Source: Int J Public Health. 2022 Jun 2;67:1604882. doi: 10.3389/ijph.2022.1604882 (PMC9200959; doi:10.3389/ijph.2022.1604882)

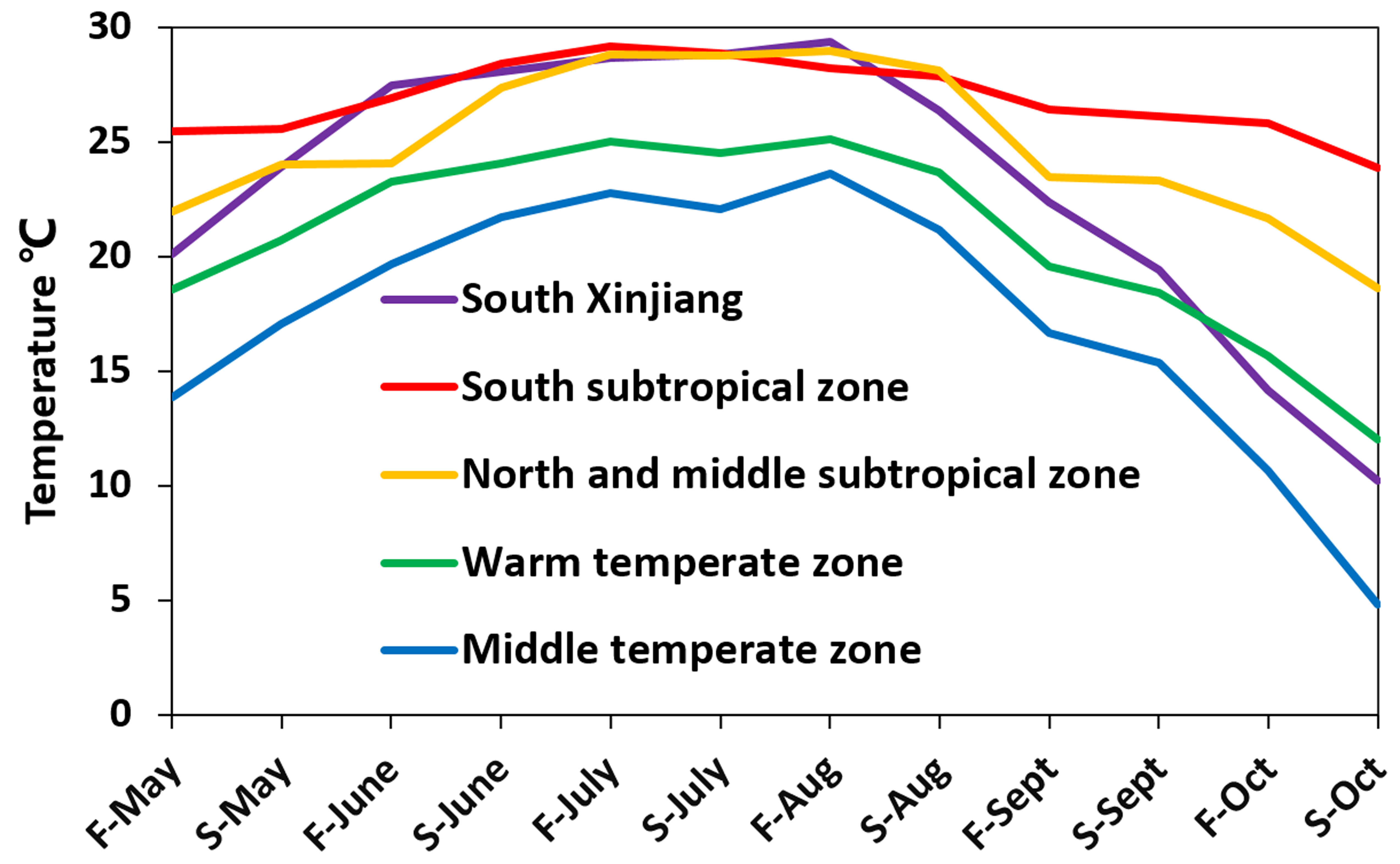

Supplement: Supplementary file 1 [file Image3.JPEG]

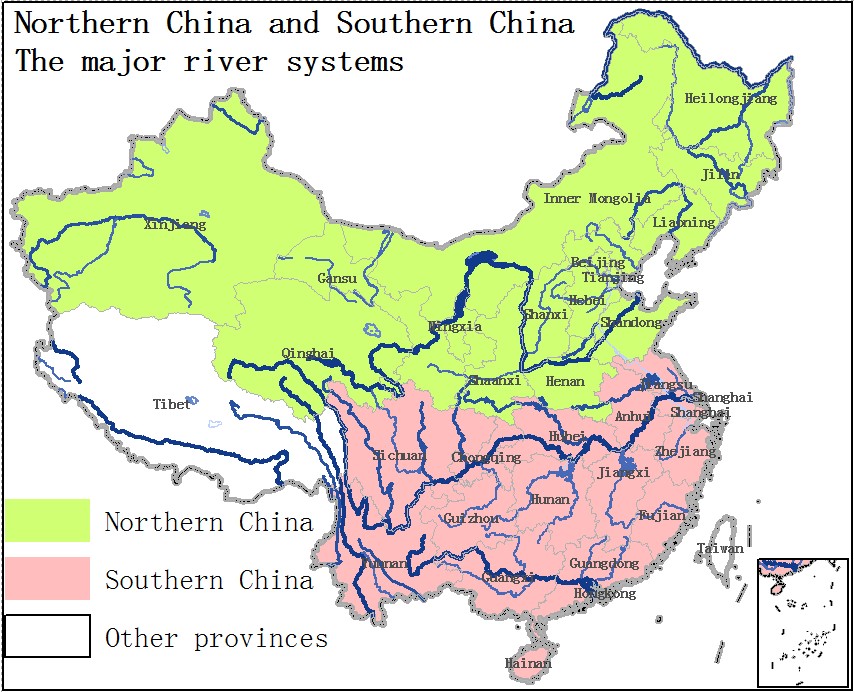

Supplement: Supplementary file 2 [file Image1.JPEG]

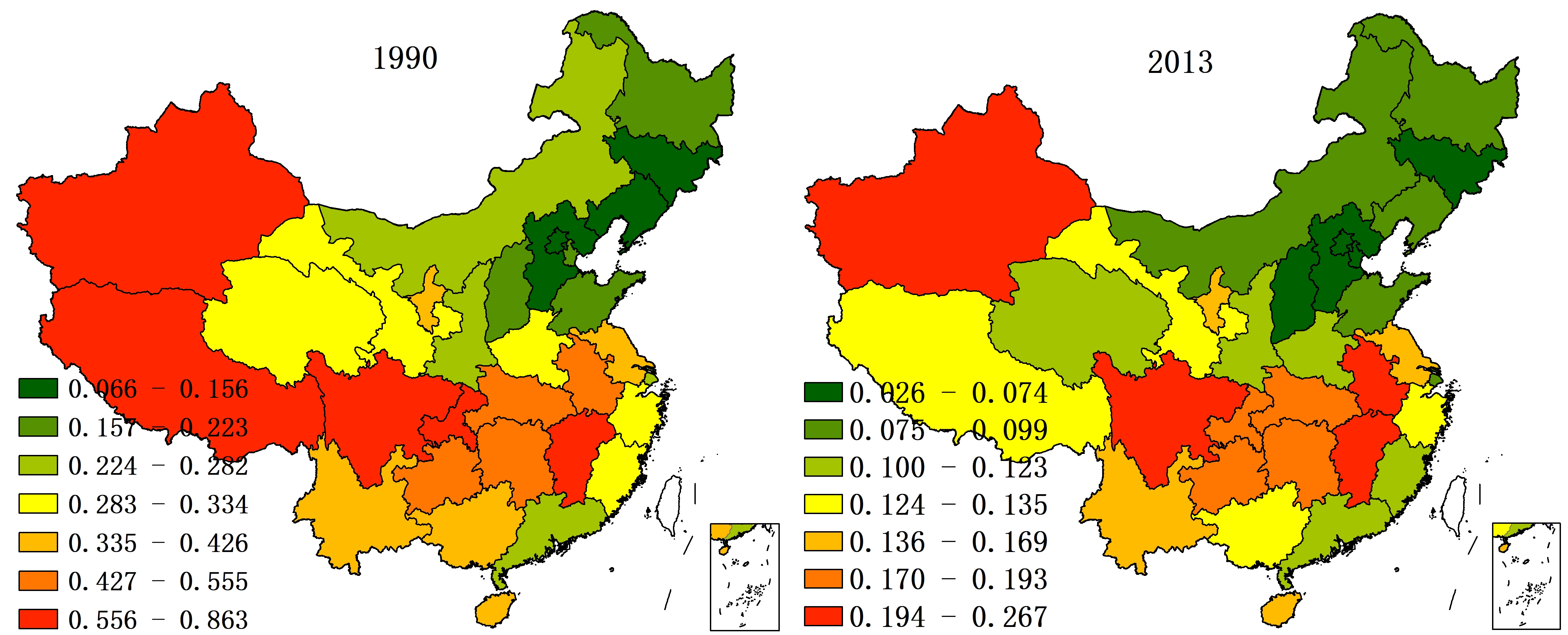

Supplement: Supplementary file 3 [file Image4.JPEG]

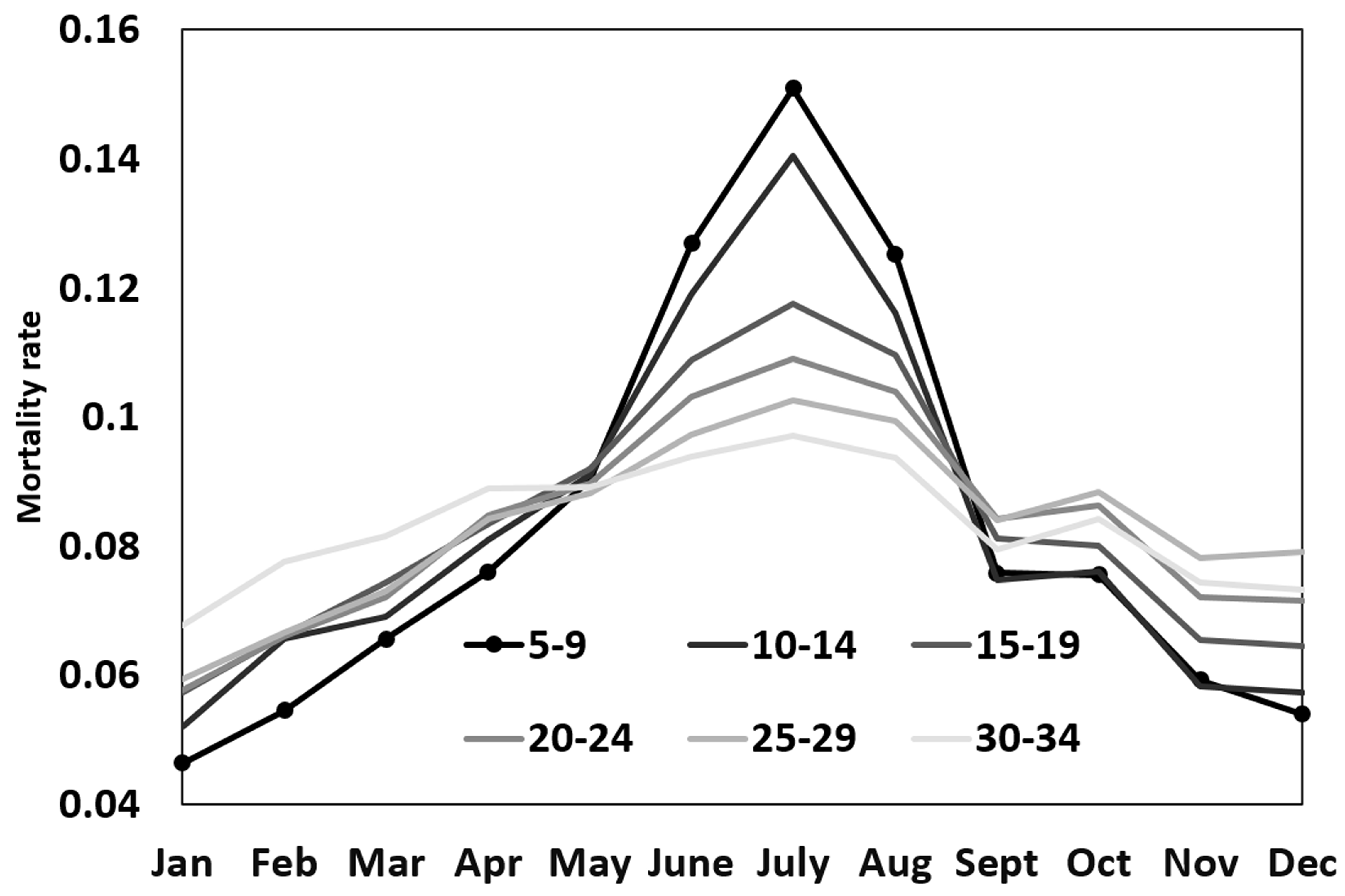

Supplement: Supplementary file 4 [file Image2.JPEG]
